# Circulating Microparticles and Coronary Plaque Components Assessed by Virtual Histology Intravascular Ultrasound of the Target Lesion in Patients with Stable Angina

**DOI:** 10.1371/journal.pone.0148128

**Published:** 2016-01-26

**Authors:** Pil-Ki Min, Minhee Cho, Sung-Yu Hong, Jong-Youn Kim, Eui-Young Choi, Young-Won Yoon, Byoung Kwon Lee, Bum-Kee Hong, Se-Joong Rim, Hyuck Moon Kwon

**Affiliations:** 1 Cardiology Division, Department of Internal Medicine, Gangnam Severance Hospital, Yonsei University College of Medicine, Seoul, Korea; 2 Severance Institute for Vascular and Metabolic Research, Yonsei University College of Medicine, Seoul, Korea; 3 Cardiovascular Product Evaluation Center, Yonsei University Health System, Seoul, Korea; Mathematical Institute, HUNGARY

## Abstract

High levels of microparticles (MPs) circulate in the blood of patients with atherosclerotic diseases where they can serve as potential biomarkers of vascular injury and cardiovascular outcome. We used virtual histology intravascular ultrasound (VH-IVUS) to evaluate the relationship between the levels of circulating MPs and the coronary plaque composition in patients with stable angina. We included 35 patients with stable angina (22 men, age 64 ± 9 years) and a de novo target lesion. Preintervention gray-scale and VH-IVUS analysis was performed across the target lesion. Volumetric analysis was performed over a 10-mm-long segment centered at the minimum luminal site. Blood samples were obtained from the femoral artery before coronary angioplasty. MPs were measured using a solid-phase capture assay from a commercial kit. We divided participants into either a low MPs group or high MPs group based on the median value of MPs. There was no significant difference in baseline characteristics between the groups. The plaque burden and remodeling index were similar between the groups. The presence of VH-IVUS-derived thin-cap fibroatheroma was not different between the groups. The percentage of the necrotic core (NC) was significantly higher in the high MPs group than in the low MPs group, both in planar (17.0 ± 8.8% vs. 24.1 ± 6.9%, *p* = 0.012) and volumetric analyses (17.0 ± 4.8% vs. 22.1 ± 4.3%, *p* = 0.002). Circulating MPs were positively correlated with the percentage of the NC area at the minimal luminal site (r = 0.491, p = 0.003) and the percentage of the NC volume (r = 0.496, p = 0.002). Elevated levels of circulating MPs were associated with the amount of NC in the target lesion in those with stable angina, suggesting a potential role of circulating MPs as a biomarker for detecting unstable plaque in patients with stable angina.

## Introduction

Microparticles (MPs) are submicron membrane vesicles that are produced by various vascular or peripheral blood cells following cellular activation or apoptosis [1]. MPs with complex procoagulant and proinflammatory properties circulate in the blood and can be accumulated in complicated atherosclerotic plaques [2]. The sequestered MPs within atherosclerotic plaques can also be exposed to circulation by plaque erosion or rupture [3]. Therefore, it has been reported that circulating MPs are elevated in patients with acute coronary syndrome (ACS) [4–6]. Recently, it has also been proposed that circulating MPs can be considered a surrogate marker of vulnerable plaques in the setting of stable angina [7] or asymptomatic carotid artery stenosis [8]. In addition, the level of circulating MPs can be an independent predictor of cardiovascular events in patients with stable coronary artery disease [9]. Thus, high levels of MPs circulate in the blood of patients with atherosclerotic diseases where they can serve as a useful biomarker of vascular injury and a potential predictor of cardiovascular outcome. However, few studies have evaluated the relationship between circulating MPs and plaque composition by imaging studies [7]. In this study, we hypothesized that circulating MPs could be a potential biomarker for detecting unstable coronary plaque. To interrogate this hypothesis, we investigated the relationship between the levels of circulating MPs and the coronary plaque composition determined by virtual histology intravascular ultrasound (VH-IVUS) in patients with stable angina.

## Materials and Methods

### Study population

The present study prospectively enrolled 35 patients with stable angina who underwent coronary intervention for a de novo target lesion at the Gangnam Severance Hospital, Yonsei University College of Medicine, Seoul, Korea. Stable angina was defined as no change in the frequency, duration, or intensity of symptoms within 6 weeks before the intervention. The target lesion was identified by the combination of exercise electrocardiographic findings, scintigraphic reversible defects, and angiographic lesion morphology. In patients who underwent multivessel intervention, the lesion with the worst diameter stenosis and more complex morphology in the territory of the scintigraphic reversible defects was selected as the target lesion for VH-IVUS analysis.

Patients who had a history of previous percutaneous coronary intervention (PCI) or coronary artery bypass graft, and those with an ostial lesion, chronic total occlusion, or unsuitable lesion for IVUS were excluded from this study. Other exclusion criteria were ACS, hemodynamic instability, chronic kidney disease, apparent infectious disease, chronic inflammatory disease, and a malignancy.

The study protocol was approved by the Institutional Review Board of the Gangnam Severance Hospital, Yonsei University of College of Medicine, and written informed consent was obtained from each patient.

### Study procedure and blood sampling

All patients were prescribed chronic aspirin (100 mg/day) and clopidogrel (75 mg/day) therapy for ≥5 days, or they received a loading dose of oral aspirin (300 mg) and clopidogrel (300 mg) prior to PCI. A loading dose of intravenous unfractionated heparin (10,000 IU) was given prior to PCI, and if necessary, a repeat bolus of heparin was administered to maintain an activated clotting time of 250–300 s during PCI. Coronary angiography was performed by conventional methods via the femoral artery. Then, balloon angioplasty and stent implantation were performed according to standard techniques. Blood samples were obtained through the femoral sheath prior to PCI to measure the MPs. All blood samples were drawn into evacuated collection tubes containing sodium citrate (0.109 M). The plasma supernatant was quickly decanted following a 15 min configuration at 1,500 × *g* at room temperature and was then again quickly centrifuged for 12 min at 13,000 × *g* at room temperature. The platelet-poor plasma containing circulating MPs obtained by double centrifugation was then stored frozen at -80°C for future analysis.

### Intravascular ultrasound and virtual histology

Pre-PCI intravascular ultrasound (IVUS) examination was performed on the target lesion prior to predilation using a 20-MHz 2.9 French IVUS catheter (Eagle Eye, Volcano Therapeutics, Rancho Cordova, CA, USA). After an intracoronary administration of 0.2 mg of nitroglycerin, the IVUS catheter was advanced >10 mm beyond the lesion and then pulled back using a motorized pullback system to a point >10 mm proximal to the lesion at 0.5 mm/s. During pullback, gray-scale IVUS was recorded, and raw radiofrequency data were captured at the top of the R wave for reconstructing the color-coded map by a VH-IVUS data recorder (Volcano Therapeutics). Gray-scale quantitative IVUS analyses included the external elastic membrane (EEM) cross-sectional area (CSA), luminal CSA, plaque and media (P&M, i.e., the EEM minus the luminal area) CSA, and plaque burden (i.e., the P&M divided by the EEM). The remodeling index for the PCI target lesion was defined as the ratio of the EEM CSA at the cross-section with a minimum luminal area to the reference EEM CSA (i.e., the average of the proximal and distal reference segments). VH-IVUS analysis color-coded tissue as dark green (fibrous tissue [FT]), light green (fibrofatty [FF]), red (the necrotic core [NC]), or white (dense calcium [DC]) [10]. Planar IVUS analysis was performed at the site of the minimal luminal CSA and at the site of the largest NC CSA. Volumetric IVUS analysis was performed along a 10-mm segment centered on the minimal luminal CSA using Simpson’s rule. VH-IVUS analyses were reported as relative amounts of the plaque area or volume. VH-IVUS-derived thin-cap fibroatheroma (VH-TCFA) was defined as a necrotic core >10% of the plaque area at the site of the minimal luminal CSA or the largest NC CSA in at least three consecutive frames without overlying fibrous tissue in the presence of >40% plaque burden [11].

### MP assay

The frozen samples were thawed just prior to analysis. MPs with procoagulant potential were measured using a solid-phase capture assay from a commercial kit (Zymuphen MP-activity kit; Hyphen BioMed, France). In brief, MPs were isolated by capture onto immobilized annexin V, and the amount of captured MPs was determined by a prothrombinase assay using their procoagulant potential. The solid-phase capture assay combined with the prothrombinase assay provides a functional assessment of the procoagulant potential of isolated circulating MPs, regardless of the capture ligand [12]. Based on the median value of MPs, we divided our participants into either a low MPs group (<the median value of MPs) or a high MPs group (≥the median value of MPs).

The cell origins of the MPs were determined by antigenic capture with insolubilized specific antibodies instead of annexin V using similar solid-phase capture methods [12]. In the present study, the following biotinylated monoclonal antibodies were used: anti-CD31, anti-CD42b (Abcam, Cambridge, UK), and anti-CD146 (Millipore, Billerica, MA, USA). Results were expressed as phosphatidylserine equivalents (PS eq), which were calculated using the standard calibration curve constructed using liposomes of known concentration. All tests were performed in duplicate.

### Statistical analysis

The normality of data was assessed using the Shapiro-Wilk normality test. Normally distributed continuous data are expressed as the mean ± standard deviation, while non-normally distributed continuous variables are presented as median and interquartile range (IQR). For reasons of uniformity, all gray-scale IVUS parameters are presented as median and IQR. VH-IVUS derived plaque components were normally distributed. Normally distributed continuous variables were analyzed using the Student’s t tests, and non-normally distributed continuous variables were compared using the Mann-Whitney U-test. Categorical data are presented as numbers and percentages. Differences in categorical variables were analyzed using the chi-square test. Correlations between continuous variables were assessed using linear regression analysis. A two-tailed *p* value < 0.05 was considered statistically significant. All statistical analyses were performed with PASW statistics, version 18.0 (SPSS, Inc., Chicago, IL, USA).

## Results

Thirty-five patients with stable angina (22 men, age 64 ± 9 years) were enrolled in this study. The enrolled patients were divided into either the low MPs group (n = 17) or high MPs group (n = 18) based on the median value of MPs (20.0 nM PS eq). In addition to the circulating MPs captured onto annexin V, the levels of circulating MPs captured with anti-CD31 or anti-CD42b were also higher in the high MPs group than in the low MPs group (Table 1). The levels of circulating MPs captured with anti-CD146 had a higher trend in the high MPs group than in the low MPs group with borderline significance (Table 1).

**Table 1:** Levels of microparticles captured on annexin V and the different monoclonal antibodies.

Captured antibody	Low MPs group (n = 17)	High MPs group (n = 18)	*p* value
Annexin V	7.7 (5.5, 9.8)	29.0 (25.9, 39.0)	< 0.001
Anti-CD31	2.6 (2.0, 5.0)	7.5 (3.8, 11.0)	0.005
Anti-CD146	1.1 (0.8, 1.7)	1.8 (1.2, 3.0)	0.060
Anti-CD42b	1.5 (0.6, 3.8)	6.1 (3.9, 9.2)	< 0.001

Data are presented as median (interquartile range) and expressed as PS eq.

Baseline clinical and biochemical characteristics were not different between the low MPs group and high MPs group as listed in Table 2. There was no difference in the pre-procedural angiographic findings between the two groups (Table 3). The left anterior descending artery was the most common target lesion location. The quantitative coronary angiographic findings were similar in both groups. The gray-scale IVUS findings are summarized in Table 4. Planar IVUS analysis performed at the site of the minimal luminal area and at the site of the largest NC area, and volumetric IVUS analysis performed along a 10-mm segment centered on the minimal luminal area were similar between the two groups. There was no significant correlation between the level of MPs and the gray-scale IVUS parameters (data not shown).

**Table 2:** Patients’ baseline characteristics.

	Low MPs group (n = 17)	High MPs group (n = 18)	*p* value
Age (years)	62 ± 10	67 ± 7	0.209
Male sex	13 (77%)	9 (50%)	0.105
Hypertension	11 (65%)	12 (67%)	0.903
Diabetes mellitus	10 (59%)	8 (44%)	0.395
Current smoker	4 (24%)	5 (28%)	0.774
Ejection fraction (%)	63.8 ± 10.5	62.7 ± 8.0	0.457
Total cholesterol level (mg/dL)	162.8 ± 39.7	154.2 ± 29.9	0.680
Triglyceride level (mg/dL)	178.3 ± 105.6	136.4 ± 74.4	0.162
HDL-cholesterol level (mg/dL)	41.2 ± 10.2	37.9 ± 7.8	0.580
LDL-cholesterol level (mg/dL)	95.5 ± 35.8	92.5 ± 26.4	0.809
White blood cell count (10^3^/μL)^a^	6.70 (5.22, 8.39)	7.49 (6.01, 8.72)	0.273
Hemoglobin level (g/dL)	13.7 ± 1.7	14.0 ± 1.5	0.729
Platelet count (10^3^/μL)	251.3 ± 59.6	268.6 ± 69.0	0.468
Glucose level (mg/dL)^a^	108 (99.5, 151.5)	102.5 (91.8, 112.8)	0.163
hs-CRP level (mg/L)^a^	1.1 (0.4, 3.1)	2.2 (0.8, 5.0)	0.081
Medication			
Aspirin	8 (47%)	12 (67%)	0.241
Beta-blocker	4 (24%)	5 (28%)	0.774
ACEI/ARB	9 (53%)	10 (56%)	0.877
Statin	9 (53%)	13 (72%)	0.238

Data are presented as the number (%) of patients or mean ± standard deviation (SD). HDL, high-density lipoprotein; LDL, low-density lipoprotein; hs-CRP, high-sensitivity C-reactive protein; ACEI, angiotensin converting enzyme inhibitor; ARB, angiotensin receptor blocker.

^a^Data are presented as median (interquartile range).

**Table 3:** Angiographic findings.

	Low MPs group (n = 17)	High MPs group (n = 18)	*p* value
Target lesion			0.384
LAD	15 (88%)	13 (72%)	
LCx	1 (6%)	1 (6%)	
RCA	1 (6%)	4 (22%)	
Number of diseased vessels			0.305
1	7 (41%)	5 (28%)	
2	8 (47%)	7 (39%)	
3	2 (12%)	6 (33%)	
Reference diameter (mm)	3.2 ± 0.4	3.3 ± 0.4	0.966
MLD (mm)	0.7 ± 0.4	0.6 ± 0.3	0.764
Diameter stenosis (%)	78.6 ± 10.3	80.6 ± 7.6	0.428
Lesion length (mm)	22.5 ± 7.5	25.4 ± 11.0	0.526

Data are presented as the number (%) of patients or mean ± SD. LAD, left anterior descending artery; LCx, left circumflex artery; RCA, right coronary artery; MLD, minimal lumen diameter.

**Table 4:** Gray-scale IVUS findings.

	Low MPs group (n = 17)	High MPs group (n = 18)	*p* value
Site of the minimum luminal area			
EEM CSA (mm^2^)	13.3 (10.1, 15.9)	13.1 (11.6, 15.3)	0.782
Lumen CSA (mm^2^)	2.8 (2.3, 3.9)	2.8 (2.3, 3.3)	0.636
P&M CSA (mm^2^)	10.2 (7.7, 13.4)	10.7 (9.0, 12.8)	1.000
Plaque burden (%)	78.5 (69.7, 82.4)	78.0 (75.0, 81.5)	0.935
Remodeling index	1.06 (1.00, 1.13)	0.98 (0.90, 1.08)	0.245
Site of the largest necrotic core			
EEM CSA (mm^2^)	14.0 (11.2, 15.9)	13.2 (12.4, 15.2)	0.935
Lumen CSA (mm^2^)	3.6 (2.8, 5.0)	3.0 (2.8, 3.9)	0.245
P&M CSA (mm^2^)	9.7 (6.9, 12.9)	10.1 (9.5, 12.4)	0.660
Plaque burden (%)	68.8 (59.3, 80.8)	76.2 (71.8, 78.3)	0.335
Remodeling index	1.09 (1.01, 1.19)	1.06 (0.91, 1.18)	0.405
Target lesion volume (10-mm segment)			
Vessel volume (mm^3^)	126.5 (112.5, 141.0)	128.3 (109.8, 155.8)	0.636
Lumen volume (mm^3^)	39.5 (17.3, 50.7)	34.6 (26.8, 52.4)	0.909
P&M volume (mm^3^)	94.5 (66.0, 117.6)	92.7 (76.6, 109.4)	1.000
Plaque burden (%)	73.8 (56.1, 84.0)	72.5 (60.6, 76.9)	0.883

Data are presented as median (interquartile range).

**Fig 1:**
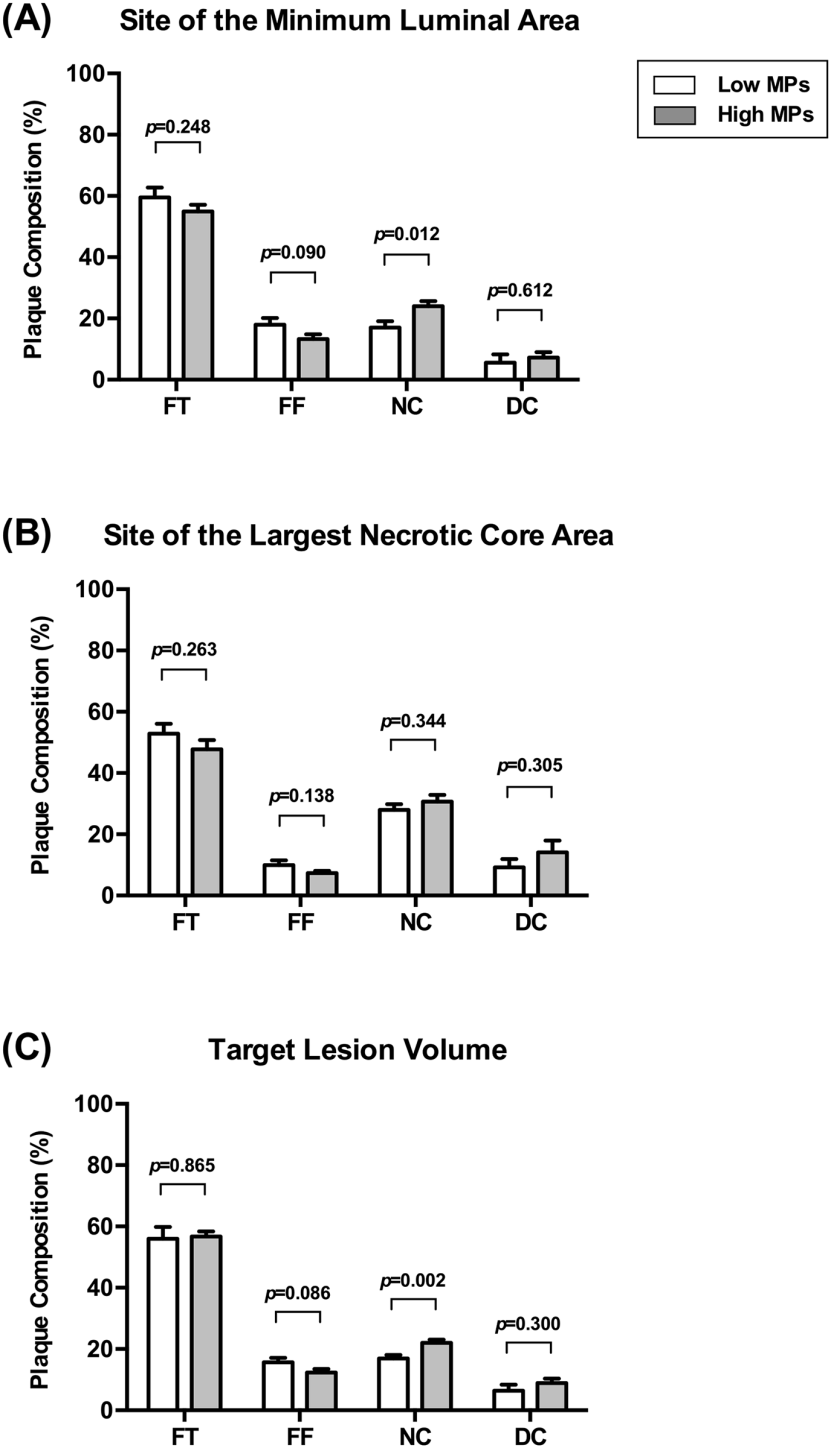
Comparison of the relative plaque composition between the two groups. The site of the minimum luminal area (A), site of the largest necrotic core area (B), and target lesion volume of a 10-mm segment centered on the minimal luminal area (C). Plaque compositions assessed by VH-IVUS were classified into 4 major components: FT, FF, NC, and DC. Data are shown as mean ± 95% confidence interval.

**Fig 2:**
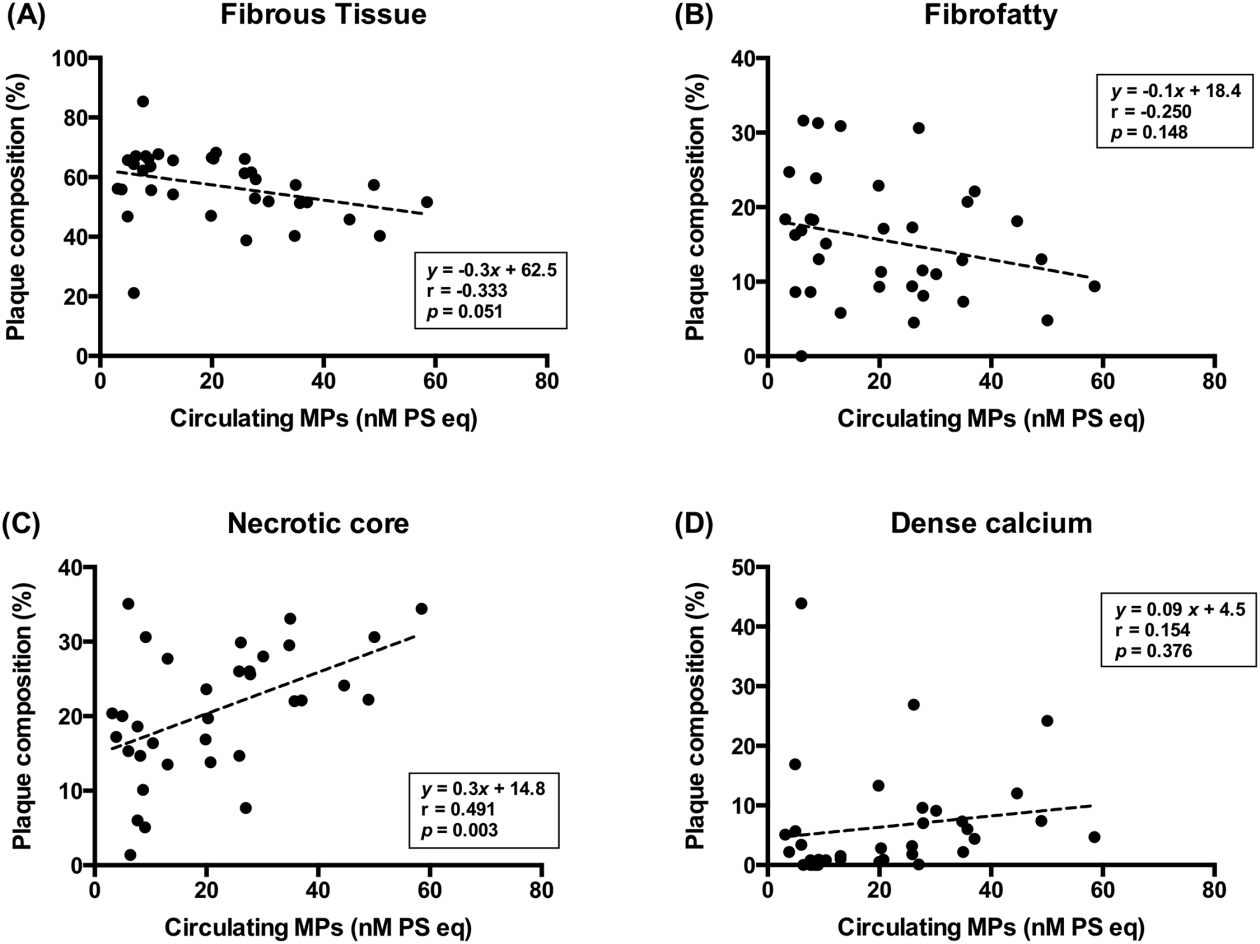
Correlation between the levels of circulating MPs and relative plaque components assessed by planar VH-IVUS. The following components at the sites of the minimal luminal area are shown: (A) FT, (B) FF, (C) NC, and (D) DC.

**Fig 3:**
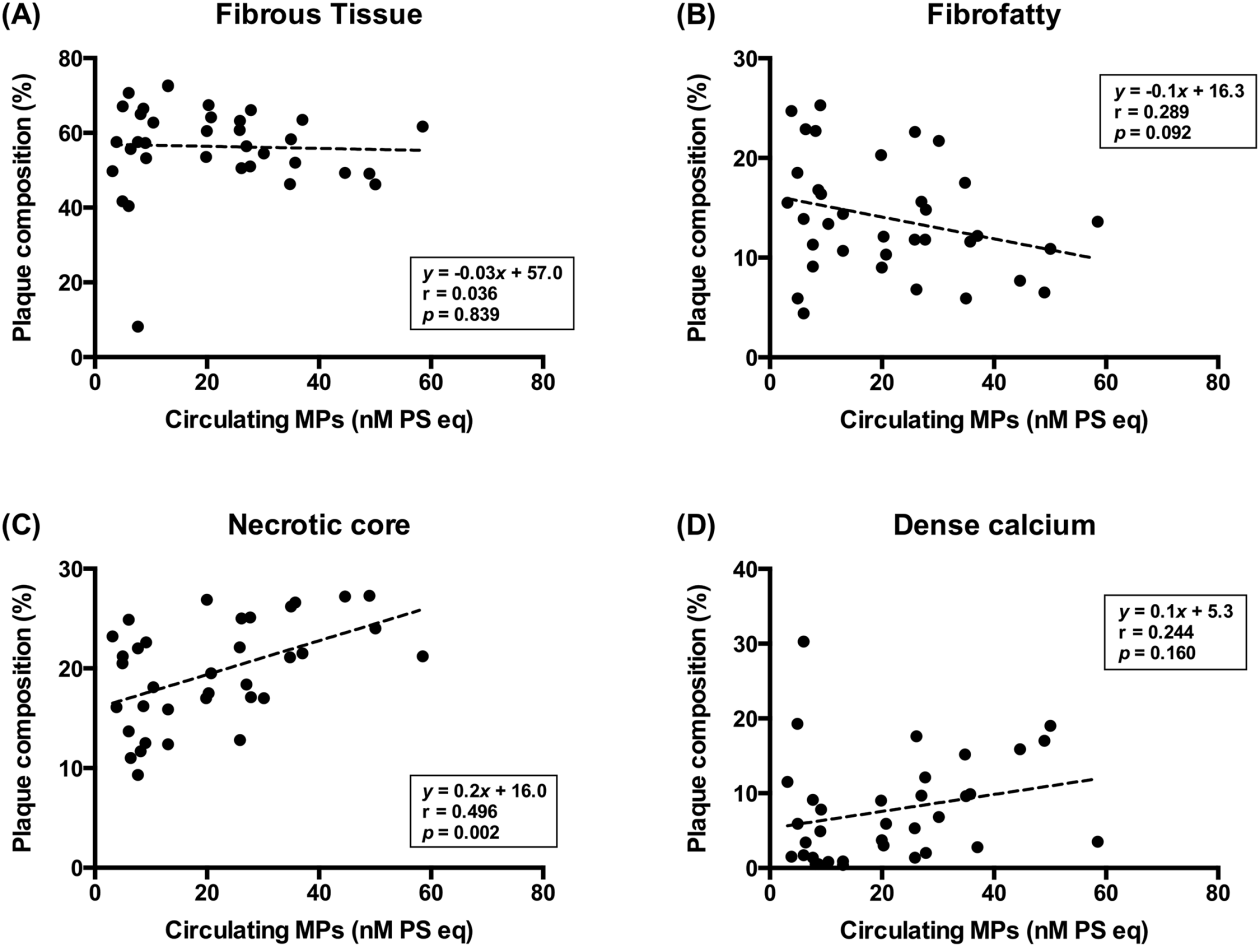
Correlation between the levels of circulating MPs and relative plaque components assessed by VH-IVUS analysis. The target lesion volume of a 10-mm segment centered on the following minimal luminal areas is shown: (A) FT, (B) FF, (C) NC, and (D) DC.

The relative plaque composition was compared between the two groups using planar and volumetric VH-IVUS analyses (Fig 1). The presence of VH-TCFA was not different between the low MPs group and high MPs group (4/17 [24%] vs. 6/18 [33%], *p* = 0.711). Although there was no difference in the percentage of FT, FF, and DC between the two groups at the site of the minimal luminal area, the percentage of the NC component was higher in the high MPs group than in the low MPs group (17.0 ± 8.8% vs. 24.1 ± 6.9%, *p* = 0.012) (Fig 1A). At the site of the largest NC area, no significant difference was observed in the plaque composition between the two groups (Fig 1B). In volumetric VH-IVUS analysis of the target lesion, the percentage of the FT, FF, and DC volume was not different between the two groups. However, the percentage of the NC volume was significantly higher in the high MPs group than in the low MPs group (17.0 ± 4.8% vs. 22.1 ± 4.3%, *p* = 0.002) (Fig 1C). Furthermore, relationships between the levels of circulating MPs and the percentage of each plaque component were analyzed using planar VH-IVUS at the site of the minimal luminal area (Fig 2) and volumetric VH-IVUS at the target lesion volume (Fig 3). The levels of circulating MPs were positively correlated with the percentage of the NC area at the minimal luminal site (r = 0.491, *p* = 0.003) (Fig 2) and the percentage of the NC volume at the target lesion (r = 0.496, *p* = 0.002) (Fig 3). When the levels of MPs captured on different monoclonal antibodies were analyzed, there was no significant correlation between the MPs levels and the percentage of each plaque component regardless of their cellular origins (data not shown).

## Discussion

In the present study, we demonstrated that the percentage of NC was significantly higher in patients with high levels of MPs in the setting of stable angina. Moreover, circulating MPs were positively correlated with the percentage of NC both in planar and volumetric analyses using an VH-IVUS study. Until now, many efforts have been made to predict high-risk lesions or vulnerable plaques using plasma biomarkers in patients with stable coronary artery disease. VH-IVUS has been frequently used to obtain detailed information about the composition and characteristics of coronary atherosclerotic plaques [13, 14]. Several plasma biomarkers have been proposed as predictors of ruptured plaque [15] or the plaque composition assessed by VH-IVUS [16, 17]. Because of their procoagulant and proinflammatory potential, extensive studies have been performed regarding the role of MPs in atherothrombotic disease such as ACS [4–6]. It has been proposed that circulating MPs can be considered as a surrogate marker of vulnerable plaque or global vascular damage [18]. Bernal-Mizrachi et al. [19] were the first to describe the correlation between circulating MPs and the angiographic lesion morphology in patients with ACS. They demonstrated that high circulating levels of endothelial MPs correlated with high-risk angiographic lesions, and suggested that circulating endothelial MPs can be a useful marker to detect endothelial injury and the risk of ACS [19]. Bernard et al. [7] also reported that circulating levels of endothelial MPs were significantly higher in the presence of non-calcified plaques assessed by multidetector computed tomography in patients with type 2 diabetes. However, the relationship between circulating MPs and plaque composition determined by VH-IVUS remains unknown. We report for the first time a relationship between the levels of circulating MPs and the coronary plaque components assessed by VH-IVUS in patients with stable angina.

We demonstrated previously that circulating MPs were elevated locally in culprit coronary arteries in patients with ST-segment elevation myocardial infarction, and suggested a potential role of circulating MPs in coronary atherothrombosis in the early period of myocardial infarction [20]. However, in the current study, the origin of elevated circulating MPs could not be determined and there was no significant correlation between the levels of MPs captured on different monoclonal antibodies and the percentage of each plaque component. Therefore, the mechanisms underlying the elevation of circulating MPs and their cellular origin remain to be elucidated. Considering the pathophysiology of stable angina, the elevated levels of circulating MPs in unstable coronary plaques with a high NC content can be caused by endothelial injury and a subsequent inflammatory process. As a surrogate marker of the inflammatory process, an elevated hs-CRP level was previously reported to be related to the plaque burden in nonculprit coronary artery [21] and the amount of NC in the culprit lesion of patients with stable angina [16]. In our study, the hs-CRP levels showed a positive correlation with the levels of circulating MPs (r = 0.467, *p* = 0.005), but we could not find any relationship between the hs-CRP levels and percentages of each plaque component (data not shown). Considering the weak correlation between the hs-CRP level and percentage of plaque components in a previous study [16], the difference in the number of the study population may explain the discrepancy between the studies. In our study, circulating MPs were a more sensitive marker than the hs-CRP level for identifying unstable plaques with a high NC content. However, the presence of VH-TCFA did not differ between the low MPs group and high MPs group. There are several reasons for the lack of a relationship between the presence of VH-TCFA and the level of MPs in the present study. First, the study sample was too small to detect a difference in the presence of VH-TCFA between the two groups. Moreover, the IVUS study was performed only in the target vessel, and presence of VH-TCFA in non-target vessels could not be evaluated in this study. Second, a follow-up IVUS was not performed in this study. Therefore, dynamic change in coronary lesions with high NC contents could not be assessed.

It has been reported that plasma MPs mainly originate from platelets in contrast to the abundant leukocyte-originating MPs within atherosclerotic plaques [3]. However, Sarlon-Bartoli et al. [8] demonstrated that plasma level of leukocyte-derived MPs was significantly higher in patients with unstable carotid plaque than in those with stable plaque. In addition, plaque MPs can participate in intercellular communication [22, 23]. Therefore, circulating MPs derived from other cells beyond platelet or endothelial cells can also participate in the formation of unstable plaques. However, whether an elevated level of circulating MPs originates from unstable plaque or whether elevated MPs have an effect on the formation of unstable plaque is yet to be determined. Moreover, the role of MPs varies depending on their origin and composition. Therefore, future work will be required to determine the origin of elevated levels of MPs and their specific biological role in the formation of unstable plaque.

The present study has some limitations. First, this was a single center study, and the study sample was relatively small. Therefore, our results need to be validated in larger studies. Second, the IVUS study was performed only in the target vessel determined by angiographic analysis. Therefore, possible influence from unstable plaques in non-target vessels could not be excluded. Third, the effect of medications, including antiplatelet agents, on the production of circulating MPs could not be analyzed in this study. Thus, we could not rule out the possible impact of medications on the level of circulating MPs. Finally, follow-up data were not available and the prognostic implication of elevated circulating MPs in patients with stable angina could not be assessed in the current study. Despite these limitations, our study addressed the possibility of a correlation between the levels of circulating MPs and the plaque composition in the target lesion of patients with stable angina.

In conclusion, elevated levels of circulating MPs were related to the amount of NC in the target lesion of stable angina. These findings suggest a potential role of circulating MPs as a biomarker for detecting unstable plaques in patients with stable angina.
